# Activated α_2_-Macroglobulin Regulates LRP1 Levels at the Plasma Membrane through the Activation of a Rab10-dependent Exocytic Pathway in Retinal Müller Glial Cells

**DOI:** 10.1038/s41598-019-49072-6

**Published:** 2019-09-13

**Authors:** Javier R. Jaldín-Fincati, Virginia Actis Dato, Nicolás M. Díaz, María C. Sánchez, Pablo F. Barcelona, Gustavo A. Chiabrando

**Affiliations:** 1 Universidad Nacional de Córdoba, Facultad de Ciencias Químicas, Departamento de Bioquímica Clínica, Córdoba, Argentina; 2 Consejo Nacional de Investigaciones Científicas y Técnicas (CONICET), Centro de Investigaciones en Bioquímica Clínica e Inmunología (CIBICI), Córdoba, Argentina; 3 Present Address: Department of Biological Sciences, University of Toronto at Scarborough, Toronto, ON Canada

**Keywords:** Retinopathy of prematurity, Protein translocation, Extracellular matrix

## Abstract

Activated α_2_-macroglobulin (α_2_M*) and its receptor, low-density lipoprotein receptor-related protein 1 (LRP1), have been linked to proliferative retinal diseases. In Müller glial cells (MGCs), the α_2_M*/LRP1 interaction induces cell signaling, cell migration, and extracellular matrix remodeling, processes closely associated with proliferative disorders. However, the mechanism whereby α_2_M* and LRP1 participate in the aforementioned pathologies remains incompletely elucidated. Here, we investigate whether α_2_M* regulates both the intracellular distribution and sorting of LRP1 to the plasma membrane (PM) and how this regulation is involved in the cell migration of MGCs. Using a human Müller glial-derived cell line, MIO-M1, we demonstrate that the α_2_M*/LRP1 complex is internalized and rapidly reaches early endosomes. Afterward, α_2_M* is routed to degradative compartments, while LRP1 is accumulated at the PM through a Rab10-dependent exocytic pathway regulated by PI3K/Akt. Interestingly, Rab10 knockdown reduces both LRP1 accumulation at the PM and cell migration of MIO-M1 cells induced by α_2_M*. Given the importance of MGCs in the maintenance of retinal homeostasis, unravelling this molecular mechanism can potentially provide new therapeutic targets for the treatment of proliferative retinopathies.

## Introduction

The low-density lipoprotein (LDL) receptor-related protein 1 (LRP1) is a member of the LDL receptor gene family. It is a cell surface glycoprotein synthesized as a precursor of 600 kDa, which is proteolytically cleaved by furin into two subunits: the 515 kDa LRP1-α subunit that contains four clusters (I-IV) of extracellular ligand-binding repeats, and the 85 kDa LRP1-β subunit, comprised of a small ectodomain, a membrane-spanning region and cytoplasmic tail^1^. Through its extracellular subunit, LRP1 binds many different ligands, including the α_2_-macroglobulin (α_2_M)-proteinase complex also known as activated α_2_-macroglobulin (α_2_M*)^2^. Recognition of α_2_M* by LRP1 requires the interaction of this ligand with the extracellular ligand-binding repeats in clusters I and II of the LRP1-α subunit^3^. In this regard, it is known that α_2_M* selectively binds only to LRP1 among all the LDL receptor family members^4^ and it is specifically internalized through clathrin-dependent endocytosis. Interestingly, during the progression of several proliferative retinal diseases such as proliferative diabetic retinopathy (PDR) and sickle cell retinopathy (SCR), increased extracellular proteolysis leads to exacerbated extracellular matrix remodeling^5,6^. This remodeling is characterized by the accumulation of proteases and their inhibitors at the lesion site, requiring their clearance for the recovery of retinal homeostasis. If homeostasis is not reestablished, the retina can be severely injured, which may culminate in neovascularization, vitreous hemorrhage, and mechanical damage such as retinal detachment^7^. LRP1 plays a key role in protecting retinal tissue through the clearance of the protease/inhibitor complexes, particularly α_2_M*. Furthermore, it has also been described that the α_2_M*/LRP1 interaction, in spite of inducing endocytosis of the inhibitor-protease complex, also activates different intracellular signaling pathways in numerous cell types, including macrophages^8–11^, Schwann cells^12^, and Müller glial cells (MGCs)^13^. In this regard, we have previously demonstrated that α_2_M* promotes MGCs migration by regulating the matrix metalloproteinases (MMPs) activity through LRP1^14^.

Under physiological conditions, MGCs expressing LRP1^14^, are extended throughout the retina and interact with almost every retinal cell type, providing crucial structural and functional support to neurons and blood vessels^15^. Nevertheless, under ischemic conditions, neovascular rat retinas exhibited augmented levels of α_2_M and increased expression of LRP1 in MGCs that is associated with enhanced activity of matrix metalloproteinase-2 (MMP-2) and MMP-9^16^. These findings were also confirmed in both the vitreous humour and retinas of patients with neovascular disorders such as PDR and SCR^17,18^. Interestingly, MGCs strongly monitor retinal environment and in response to retinal imbalance increase both gene and protein expression levels of fibrillary acidic protein (GFAP)^19–23^. In this sense, we have demonstrated that α_2_M* is also able to induce GFAP expression via LRP1 in Moorfields/Institute of Ophthalmology-Muller 1 (MIO-M1) cells^13^. These results were also confirmed in an animal model, by the intravitreal injection of α_2_M at a similar concentration to those reported in diabetic patients, which highlight that both α_2_M and LRP1 are involved in the activation of MGCs during ischemic proliferative diseases^13^. However, the mechanism whereby α_2_M and LRP1 participate in the aforementioned pathologies is not well established.

It is widely assumed that after endocytosis LRP1 recycles back to the plasma membrane (PM) while its ligands reach different intracellular fates. For instance, α_2_M* follows a lysosomal degradation pathway^24,25^, whereas apolipoprotein E (ApoE) and gp96 heat shock protein avoid intracellular degradation and are instead re-exocytosed^26,27^. Whether or not the intracellular distribution of LRP1 is modified by interactions with its ligands has not been fully examined. On the other hand, it is known that LRP1 regulates the abundance of several receptors in the PM involved in cell motility and migration, such as platelet-derived growth factor receptor β (PDGFRβ)^28^, urokinase-plasminogen activator receptor (uPAR)^29^, and β1-integrin^11,30^. Accordingly, we have reported that α_2_M* regulates the endocytosis and subsequent recycling of the membrane-type matrix metalloproteinase 1 (MT1-MMP) to the cell surface, which promotes MMP-2 activation and cell migration of MIO-M1 cells^14^. Surprisingly, while MT1-MMP recycling to the PM depends on Rab11 activation, we did not observe significantly higher levels of colocalization between Rab11 and LRP1 after α_2_M* stimulation in MIO-M1 cells, which could indicate that a different pathway is responsible for the return of LRP1 to the PM. Recently, we showed that LRP1 is stored in small perinuclear vesicles (of approximately 100 nm diameter) in MIO-M1 cells, which we termed LRP1 storage vesicles (LSVs).These LSVs can mediate the insulin-regulated exocytosis of this receptor through the activation of Rab8A and Rab10^31^. However, whether these Rab-GTPases are also implicated in LRP1 sorting to the PM after α_2_M* stimulation is unknown. Unravelling this molecular mechanism is paramount, as LRP1 levels at the PM may be associated with MGCs activation and migration, representing a potential therapeutic target for retinopathies. In this regard, we hypothesized that the intracellular and cell surface distribution of LRP1 in MGCs determines their ability to interact with α_2_M* and trigger the molecular mechanisms leading to intracellular signaling activation and cell migration.

Considering all the above, herein we investigate whether α_2_M* regulates both the intracellular distribution and sorting of LRP1 to the PM and the molecular mechanisms whereby this regulation is involved in the cell migration of MIO-M1 cells.

## Results

### α_2_M* induces LRP1 accumulation in early, but not in late endosomes or acidic/degradative compartments of MIO-M1 cells

**Figure 1:**
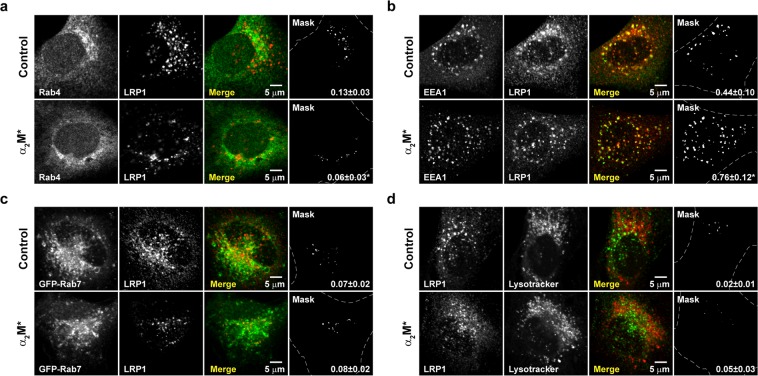
α_2_M* induces LRP1 intracellular redistribution in MIO-M1 cells. The cellular distribution of LRP1, in untreated or α_2_M* treated (60 nM for 30 min) MIO-M1 cells, was compared to that of (**a**) Rab4-positive endosomes, (**b**) EEA1-positive early endosomes, (**c**) GFP-Rab7-positive late endosomes, and (**d**) LysoTracker-stained vesicles. Representative confocal micrographs (middle optical section planes) are shown, along with merge images showing the colocalization of LRP1 (red in a–c and green in d) with each specific intracellular marker (green or red as appropriate). The mask images depict colocalized pixels (white) in merge images and the mean of the Manders’ coefficients with their respective standard deviations (MC ± SD). Three independent experiments were performed and at least 50 cells were analyzed per condition. Asterisks (*) indicate significant differences (*P* < 0.05) relative to control.

Although it is known that LRP1 mediates the endocytosis and subsequent lysosomal degradation of α_2_M*, the intracellular distribution of the receptor after this ligand-receptor interaction is poorly characterized. Accordingly, we first evaluated the subcellular localization of LRP1 in MIO-M1 cells before and after stimulation with α_2_M*. Using confocal microscopy, LRP1 was immunodetected in Rab4-positive endosomes in non-stimulated cells (Manders’ coefficient, MC = 0.13 ± 0.03). Remarkably, this level of colocalization was significantly decreased by the presence of α_2_M* (MC = 0.06 ± 0.03) (Fig. 1a). On the other hand, the proportion of LRP1 in EEA1-positive early endosomes (MC = 0.44 ± 0.10) was significantly increased after stimulation with α_2_M* (MC = 0.76 ± 0.12) (Fig. 1b). Conversely, LRP1 was sparsely distributed in Rab7-positive late endosomes or acidic/degradative compartments (LysoTracker-stained vesicles), which was unchanged by stimulation with α_2_M* (MC = 0.07 ± 0.02 vs 0.08 ± 0.02 and MC = 0.02 ± 0.01 vs 0.05 ± 0.03, for late endosomes and degradative compartments, respectively) (Fig. 1c,d). Importantly, we have previously demonstrated that a proportion of LRP1 is localized in Rab11-positive recycling endosomes in MIO-M1 cells (~20%), which is not significantly modified after stimulation with α_2_M*^14^. Altogether, these results indicate that α_2_M* induces both the accumulation of LRP1 in early endosomes and its depletion in Rab-4 positive vesicles, without altering the proportion of the receptor in Rab11-positive recycling endosomes and acidic/degradative compartments.

### α_2_M* is degraded by lysosomes after LRP1-mediated internalization in MIO-M1 cells

**Figure 2:**
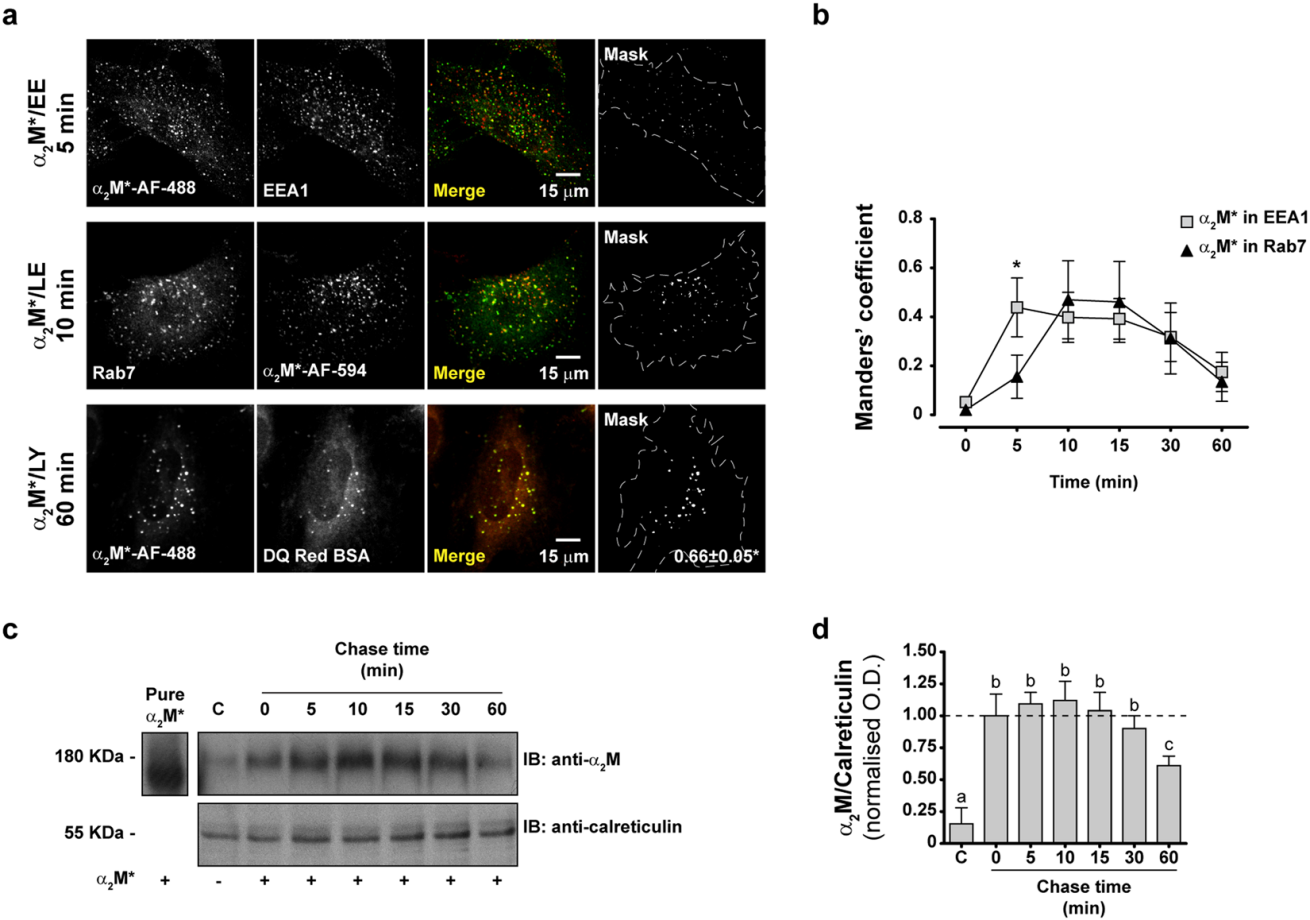
α_2_M* is degraded by lysosomes after internalization in MIO-M1 cells. (**a**) Representative confocal micrographs (middle optical section planes) of MIO-M1 cells showing the intracellular distribution of either α_2_M*-AF-488 or α_2_M*-AF-594 along with that for EEA1-positive early endosomes (EE), Rab7-positive late endosomes (LE), or lysosomes labeled with fragmented DQ-Red BSA (LY) during pulse-chase experiments with chase time points at 0, 5, 10, 15, 30, and 60 min. Merge images show the colocalization of α_2_M*-AF-488 (green) or α_2_M*-AF-594 (red) with each specific intracellular marker (green or red as appropriate). The mask images depict colocalized pixels (white) in merge images and, for DQ-Red BSA, it is also showing the mean of the Manders’ coefficients with its respective standard deviations (MC ± SD). Three independent experiments were performed and at least 25 cells were analyzed per condition. (**b**) Dot-plot graph showing the means of the Manders’ coefficients with their respective SD for the colocalization of fluorescent α_2_M* conjugates with early or late endosomes during the aforementioned pulse-chase experiments. Asterisk indicates significant differences (*P* < 0.05) between MC at one specific time point. (**c**) Representative immunoblot (IB) image showing internalized α_2_M* in whole lysates of MIO-M1 cells that were incubated with the ligand during pulse-chase experiments with chase time points at 0, 5, 10, 15, 30, and 60 min. Calreticulin was used as a loading control and pure α_2_M* (10 µg) or the whole lysate of untreated MIO-M1 cells (C) was loaded as antibody specificity controls (positive and negative controls, respectively). *Inset*: short exposure image of the region corresponding to the pure α_2_M*. (**d**) Bar graph showing the relative α_2_M in MIO-M1 cells calculated as the ratio of α_2_M/calreticulin relative to chase time 0 min. Different letters indicate significant differences relative to other conditions (*P* < 0.05, n = 3), where **a** indicates significant difference compared to **b** and **c,** **b** indicates significant difference from both **a** and **c**, and **c** indicates significant difference from both **a** and **b**.

To determine the intracellular fate of α_2_M* after the interaction with LRP1 in MIO-M1 cells, we performed pulse-chase experiments with fluorescent α_2_M* conjugates (α_2_M*-Alexa Fluor-488 (α_2_M*-AF-488) or α_2_M*-Alexa Fluor-594 (α_2_M*-AF-594)) to track its intracellular route through different compartments labelled with fluorescent antibodies or probes. Using confocal microscopy, we showed that fluorescent α_2_M* conjugates move sequentially from EEA1-positive early endosomes to Rab7-positive late endosomes before finally arriving at the lysosomes (labelled with fluorescent fragments of degraded DQ-Red BSA) (Fig. 2a). Time-course analysis of the distribution of fluorescent α_2_M* conjugates in early and late endosomes (Suppl. Fig. S1) showed that the maximal accumulation of α_2_M* in the former occurred 5 min after internalization, while the accumulation in the latter peaked after 10 min (Fig. 2b). Moreover, α_2_M* degradation was supported by immunoblotting of whole cell lysates of the MIO-M1 cells that were previously incubated with the ligand during pulse-chase experiments, where we observed α_2_M* degradation after 60 min of internalization (Fig. 2c,d). As expected, pulse-chase experiments of both α_2_M*-AF-488 and transferrin-Alexa Fluor-594 (Tf-AF-594) revealed segregated pathways of internalization for both proteins (Suppl. Fig. S2). These results support the concept that the α_2_M*-LRP1 complex is internalized by endocytosis and rapidly accumulated in early endosomes, which constitutes a bifurcation point of the internalization pathways: α_2_M* proceeds to be degraded by lysosomes, while the majority of LRP1 accumulates in endocytic compartments.

### α_2_M* induces the sorting of LRP1 to the PM of MIO-M1 cells

**Figure 3:**
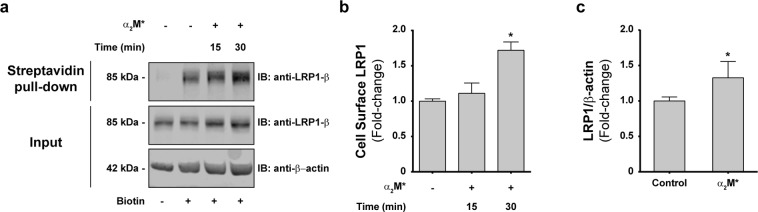
The LRP1 PM levels increase in MIO-M1 cells upon stimulation with α_2_M*. (**a**) Representative immunoblot (IB) images showing total and surface levels of LRP1-β in whole lysates of MIO-M1 cells before and after stimulation with α_2_M* (60 nM) for 15 and 30 min. Biotinylated cell surface fractions were pulled-down with streptavidin-agarose beads as described in Methods. Inputs represent 10% of total protein mass incubated with beads and β-actin was used as loading control. (**b**) Bar graph showing the proportion of LRP1 at the PM of MIO-M1 cells (relative to unstimulated cells) after stimulation with α_2_M* for 15 and 30 min. Asterisk indicates significant differences from untreated control (*P* < 0.05, n = 3). (**c**) Bar graph showing the proportion of LRP1 at the PM of MIO-M1 cells (relative to unstimulated cells) after stimulation with α_2_M* (60 nM) for 30 min. LRP1 cell surface levels were measured using a cellular ELISA for detection of PM antigens in non-permeabilized cells (antibody anti-LRP1-β). β-actin was used as loading control. Asterisk indicates significant differences relative to control (*P* < 0.05, n = 3).

**Figure 4:**
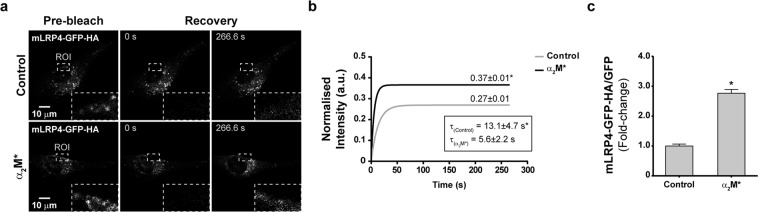
α_2_M* promotes the sorting of LRP1 to the PM mainly through exocytic pathways in MIO-M1 cells. (**a**) Representative micrographs of FRAP experiments on peripheral regions of MIO-M1 cells overexpressing mLRP4-GFP-HA, before and after stimulation with α_2_M* (60 nM) for 30 min. Photo-bleaching was performed using a region of interest (ROI) of 40 × 70 pixels (dashed-lines-squares). The ROI were digitally magnified (4x) and overlapped at the right-lower corner of each micrograph (**b**) Representative FRAP curves showing the relative fluorescence intensity recovery of mLRP4-GFP-HA over time (266.6 s). (**c**) Bar graph showing the proportion of mLRP4-GFP-HA at the PM of transfected MIO-M1 cells (relative to unstimulated transfected-cells) after stimulation with α_2_M* (60 nM) for 30 min. mLRP4-GFP-HA cell surface levels were measured using a cellular ELISA for detection of PM antigens in non-permeabilized cells (antibody anti-HA tag). Total intensity of green fluorescence protein (GFP) was used as loading control. Asterisk indicates significant differences from untreated control (*P* < 0.05, n = 3).

Considering that the presence of LRP1 at the PM determines its capability to interact with extracellular ligands promoting cell signaling, cell migration and proliferation, we decided to evaluate whether α_2_M* induces changes in the LRP1 levels at the cell surface. Accordingly, we performed biotinylation of the PM proteins followed by streptavidin pulldown and LRP1 immunoblotting in whole cell lysates of MIO-M1 cells stimulated with α_2_M*. We showed that incubations with the ligand for 30 min induced a significant increase in biotinylated-LRP1 levels at the PM (Fig. 3a,b). Moreover, similar results were obtained when the LRP1 levels at the cell surface were measured using a cellular ELISA for detection of PM antigens in non-permeabilized cells (Fig. 3c). These findings support the concept that α_2_M* induces an increase of LRP1 levels at the PM of MIO-M1 cells. However, it remains unknown whether this increase occurs as a consequence of mobilization of the receptor from storage vesicles or from recycling endosomes. Accordingly, one of the main objectives of this study was to resolve whether the accumulation of LRP1 on PM in response to α_2_M* derives from exocytic or recycling pathways. More specifically, we wanted to determine whether the interaction of LRP1 with α_2_M* at the cell surface was capable of influencing exocytosis of the intracellular pool of LRP1. For this purpose, we used the truncated mini-receptor (mLRP4-GFP-HA) that is incapable of binding α_2_M* (because it lacks clusters I and II of the α-chain required for binding)^3^. Using the mini-receptor as a surrogate reporter of the behavior of non-ligand bound receptors, we hypothesized that if α_2_M*/LRP1 interaction can influence non-ligand bound receptors, the distribution of the mini-receptor may be responsive to α_2_M* stimulation (even though it cannot bind α_2_M* itself). For that purpose, MIO-M1 cells were transiently transfected with mLRP4-GFP-HA, which generates a truncated version of the receptor. Thus, we evaluated whether α_2_M* induces changes in the kinetic of diffusion of this mini-receptor (using fluorescent recovery after photobleaching microscopy, FRAP) and whether it promotes its sorting to the PM. Figure 4a shows a representative FRAP experiment indicating the region of the cell where the photobleaching was performed (in close proximity to the cell periphery) in control and α_2_M*-stimulated MIO-M1 cells. The quantitative analysis of the FRAP experiments demonstrated that α_2_M* promoted a higher (0.37 ± 0.01 vs 0.27 ± 0.01) and faster (13.1 ± 4.7 s vs 5.6 ± 2.2 s) fluorescence recovery of mLRP4-GFP-HA in cells treated with α_2_M* compared to control conditions (Fig. 4b). Our results indicate that α_2_M* can induce the mobilization of LRP1 pools other than those that interact with the ligand at the PM. In order to investigate whether this LRP1 traffic induced by α_2_M* involves its sorting to the PM, we measured cell surface levels of mLRP4-GFP-HA by HA-tag immunodetection in transfected and non-permeabilized MIO-M1 cells. Figure 4c shows that α_2_M* promoted a significant increase in the levels of mLRP4-GFP-HA at the cell surface. Therefore, these results support the idea that α_2_M* regulates the intracellular mobilization and subsequent sorting of LRP1 to the PM mainly through exocytosis in MIO-M1 cells.

### α_2_M* induces the activation of Akt and Rab10 in MIO-M1 cells, which leads the sorting of LRP1 to the PM

**Figure 5:**
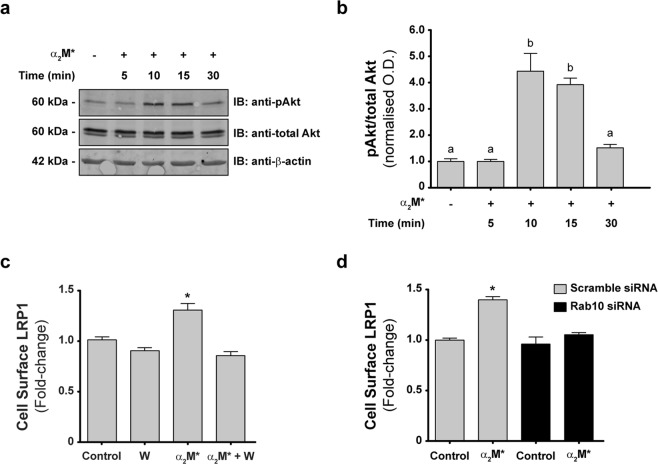
The sorting of the LRP1 to the PM depends on PI3K and Rab10 activation in MIO-M1 cells. (**a**) Representative immunoblot (IB) images showing Akt phosphorylation in whole lysates of MIO-M1 cells stimulated with either vehicle (−) or α_2_M* (60 nM) for different time points (5, 10, 15, and 30 min). Total-Akt and β-actin were used as loading controls. (**b**) Bar graph showing the relative Akt phosphorylation calculated as the ratio of p-Akt/total-Akt and expressed relative to values in control conditions. Different letters indicate significant differences relative to other conditions (*P* < 0.05, n = 3), where **b** is significantly different from **a**. (**c**) Bar graph showing the proportion of LRP1-β at the PM of MIO-M1 cells treated with vehicle (Control) or α_2_M* (60 nM) for 30 min, in absence or of presence 40 µM wortmannin (W). LRP1- β cell surface levels were measured using a cellular ELISA for detection of PM antigens in non-permeabilized cells (antibody anti-LRP1- β). β-actin was used as loading control. Asterisk indicates significant differences from untreated control (*P* < 0.05, n = 3). (**d**) Bar graph showing the proportion of LRP1-β at the PM of MIO-M1 cells transfected with either scramble siRNA or Rab10 siRNA before and after stimulation with α_2_M* (60 nM) for 30 min. LRP1- β cell surface levels were measured using a cellular ELISA for detection of PM antigens in non-permeabilized cells (antibody anti-LRP1- β). β-actin was used as loading control. Asterisk indicates significant differences relative to control of scramble siRNA (*P* < 0.05, n = 3).

Different reports have demonstrated that α_2_M* induces a variety of intracellular signaling pathways through LRP1 in several cell types^8,9,13,32^. Moreover, we have recently shown that insulin promotes LRP1 sorting to the PM of MIO-M1 cells through a process that requires the intracellular activation of the PI3K/Akt axis and the Rab-GTPases Rab8A and Rab10^31^. Taking this evidence into consideration, we evaluated whether α_2_M* induces Akt phosphorylation (p-Akt) in MIO-M1 cells. Figure 5a,b shows that p-Akt was significantly increased in MIO-M1 cells treated with α_2_M* for 10 and 15 min. Next, we examined whether this α_2_M*-induced Akt phosphorylation mediates LRP1 sorting to the PM. For that purpose, we inhibited Akt activation in MIO-M1 cells by pre-treatment with wortmannin (a PI3K inhibitor). Notably, pre-treatment with wortmannin significantly reduced the LRP1 sorting to the cell surface induced by α_2_M* (Fig. 5c). Similar results were obtained with another PI3K inhibitor, LY-294002 (data not shown). Together, these findings demonstrate that the sorting of LRP1 to the PM induced by α_2_M* requires the activation of the PI3K/Akt signaling pathway.

In previous studies, we found that LRP1 does not accumulate in Rab11-positive recycling endosomes upon stimulation with α_2_M*^14^. These unexpected results led us to hypothesize that other Rab GTPases are involved in the sorting of LRP1 to the PM. In this regard, we have also previously demonstrated that LRP1 cell surface levels in MIO-M1 cells are maintained by insulin-regulated exocytosis that requires Rab10 activation^31^. To examine the participation of Rab10 in the sorting of LRP1 to the PM, we evaluated the ability of α_2_M* to induce LRP1 sorting to the PM in cells treated with siRNA against Rab10 or negative control siRNA (scramble siRNA). Figure 5d shows that Rab10 knockdown in MIO-M1 cells significantly reduces the sorting of LRP1 to the PM^31^. Thus, our data demonstrate that Rab10 is required for the α_2_M*-induced sorting of LRP1 to the cell surface.

### The sorting of LRP1 to the PM induced by α_2_M* promotes cell migration of MIO-M1 cells

**Figure 6:**
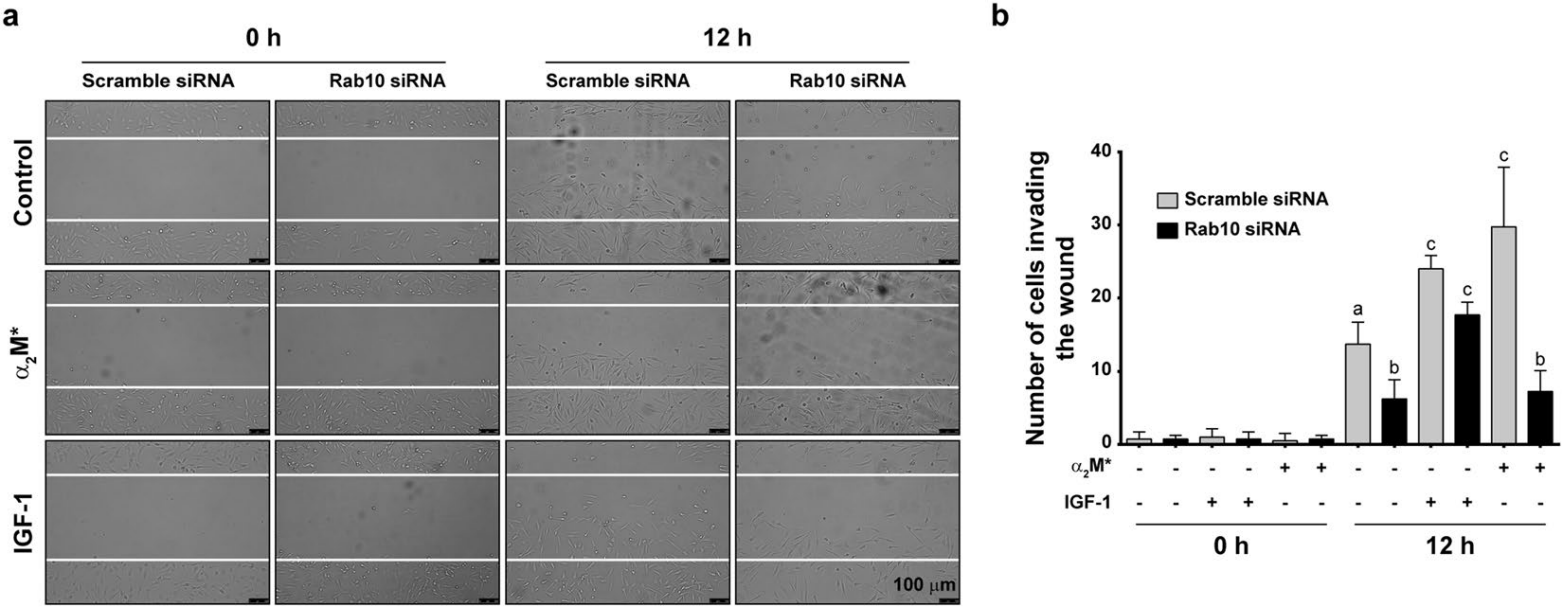
MGC migration induced by α_2_M* is impaired in Rab10-silenced cells. (**a**) Representative micrographs (DIC images) of wound-scratch assays of MIO-M1 cells transfected with either scramble siRNA or Rab10 siRNA and treated with vehicle (PBS), α_2_M* (60 nM) or IGF-1 (10 nM) for 12 h. (**b**) Bar graph showing the mean values of the number of MIO-M1 cells invading the wound (±SEM) after 12 h of treatment as described above. Different letters indicate significant differences relative to vehicle conditions for scramble siRNA or Rab10 siRNA (*P* < 0.05, n = 3), where **a** indicates significant difference compared to **b** and **c**, **b** indicates significant difference from both **a** and **c,** and **c** indicates significant differences from both **a** and **b**.

We previously reported that the α_2_M*/LRP1 interaction induces cell migration of MIO-M1 cells^14^. Based on those results and the findings of the present study, we examined whether this phenomenon depends on the increase of the LRP1 levels at the PM. Considering that Rab10 knockdown effectively reduced the sorting of LRP1 to the cell surface, we performed two-dimensional wound scratch assays (to evaluate cell migration) on MIO-M1 cells treated with siRNA against Rab10 or negative control siRNA (scramble siRNA) and stimulated with α_2_M* for 12 h as previously described^14^. Unstimulated, Rab10-silenced MIO-M1 cells had significantly impaired cell migration compared to scramble siRNA transfected cells under the same conditions. As expected, cells transfected with scramble siRNA showed a significant increase in the number of cells invading the wound after stimulation with α_2_M*. However, Rab10-silenced cells did not respond to the stimulus, whereby the amount of cells that migrated to the wound was not different from unstimulated controls (Fig. 6a,b). Notably, cell migration induced by insulin-like growth factor 1 (IGF-1) in Rab10-silenced cells was not significantly altered compared to control cells^33^, indicating a specific defect in α_2_M*-mediated migration. Taken together, these results provide evidence that the Rab10-dependent sorting of LRP1 to the PM is a key process for the cell migration of MIO-M1 cells upon α_2_M* stimulation.

## Discussion

In the present study, we characterize the molecular mechanism responsible for LRP1 sorting to the PM upon α_2_M* stimulation and how this biological process impinges on MGC migration. Our results demonstrate that, in MIO-M1 cells, the α_2_M*/LRP1 complex is internalized by endocytosis and rapidly reaches early endosomes, from which both proteins are segregated into different intracellular compartments. While α_2_M* is routed to acidic/degradation vesicles (such as late endosomes and lysosomes), LRP1 is accumulated in the PM. Although a proportion of the receptors that reach the cell surface can be sorted there from early endosomes, our results strongly suggest that exocytosis from intracellular storage compartments is the main pathway by which LRP1 enriches on the PM in response to α_2_M*. Notably, α_2_M*-dependent LRP1 sorting to the PM requires both PI3K/Akt signaling and Rab10 activation, as LRP1 enrichment on the PM is reduced by both pre-treatment with PI3K inhibitors and Rab10 knockdown. Moreover, silencing of Rab10 also impairs α_2_M*-induced cell migration, ascribing LRP1 accumulation at the PM a key role in this fundamental cellular process.

Several studies have demonstrated that different ligands of LRP1 are internalized by clathrin-dependent endocytosis and accumulate into early endosomes^34^. Most of these ligands are subsequently routed to lysosomes for degradation. However, select ligands (among which are apolipoprotein E and gp96 heat shock protein) avoid intracellular degradation and are instead re-exocytosed^26,27^. Early studies revealed that iodinated α_2_M* is among the former and is completely trafficked to acidic/degradation compartments, unlike iodinated transferrin which is accumulated in recycling endosomes and eventually re-secreted to the extracellular medium^24^. Conversely, although LRP1 also reaches early endosomes after internalization of α_2_M*, the receptor does not localize in acidic compartments, suggesting it is not degraded by lysosomes in MIO-M1 cells. Regarding other possible mechanisms of degradation of this receptor, there are previous reports demonstrating that LRP1 is mainly degraded by the proteasome after its ubiquitination^35,36^. On the other hand, it has been demonstrated that LRP1 is an important component of the glucose transporter 4 (GLUT4) storage vesicles (GSVs)^37^. Interestingly, GLUT4 can be retrieved from lysosomes through its molecular interaction with retromer^38^, increasing its stability in GSVs^38^. This rescue of GLUT4 by retromer involves the interaction with the luminal Vps10p domain of sortilin. Recently, we demonstrated that LRP1 is highly accumulated in LSVs (similar to GSVs) which can regulate the insulin-induced exocytosis of this receptor in MIO-M1 cells^31^. Interestingly, among other membrane proteins, these LSVs also contain sortilin, however, further studies are needed to demonstrate whether retromer and sortilin are involved in the putative LRP1 stabilization induced by α_2_M* in these specialized intracellular compartments.

All the LDL receptor family members, including LDL-R, recycle back to the PM through a Rab11-dependent recycling pathway^39^. In a previous study, we observed that α_2_M* can induce the Rab11-dependent recycling of MT1-MMP, but not the accumulation of LRP1 in Rab11-positive recycling endosomes^14^. On the other hand, it is known that LRP1 activity is regulated by some hormones and growth factors. Earlier studies in adipocytes revealed that insulin regulates LRP1 sorting to the PM and increases its endocytic rate^40^. This regulation is a consequence of insulin action on glucose uptake, as LRP1 is an integral protein of GSVs^37^. In this way, dynamic studies of GLUT4 recycling in adipocytes suggest that, upon insulin stimulation, LRP1 is sorted from a Rab10-independent constitutive route into a highly regulated Rab10-dependent pathway^41^. It is noteworthy that in MIO-M1 cells, insulin can likewise induce LRP1 exocytosis in a Rab11-independent manner^31^. Particularly, we demonstrated that the intracellular traffic of LRP1 induced by insulin depends on Rab8A and Rab10 and requires PI3K/Akt signaling activation. Herein, we found that α_2_M* causes intracellular redistribution of LRP1, increasing its cell surface levels in MIO-M1 cells. The intracellular traffic itinerary of this receptor induced by α_2_M* is observed with both the constitutive full-length form of the receptor and a transiently transfected mini-receptor (mLRP4-GFP-HA) version that cannot bind α_2_M*. Interestingly, α_2_M*-induced mobilization of the mini-receptor might be related to the signaling activated by α_2_M* interactions with the native form of LRP1. These findings suggest that α_2_M* induces LRP1 sorting to the PM via regulated exocytosis from intracellular vesicles, potentially LSVs, rather than the classical and well described Rab11-dependent recycling pathway. Accordingly, we demonstrated that LRP1 sorting to the PM induced by α_2_M* in MIO-M1 cells is mediated by PI3K/Akt and Rab10 activation, which is reminiscent of the mechanism previously described for LRP1 insulin-regulated exocytosis^31^. However, further studies are necessary to demonstrate whether: i) the intracellular traffic of LRP1 induced by α_2_M* and insulin also involves other Rab-GTPases associated with regulated exocytosis (such as Rab8A and Rab13); and ii) whether LSVs are the main source of LRP1 for its membrane accumulation after stimulation with both α_2_M* and insulin.

In the retina, α_2_M* and LRP1 play a key role in regulating extracellular proteolysis, by inactivation and removal of proteases from the interstitial space. This process is particularly important during the progression of proliferative disorders as protease clearance prevents neovascularization^17,18^. However, this protective effect is opposed by the fact that the α_2_M*/LRP1 interaction also causes MGC activation during the proliferative stage inducing the up-regulation of GFAP^13^, which contribute to retinal function loss and neuronal cell death^42^. In this complex scenario, MGCs become particularly relevant due to their latent stem cell potential, positioning MGCs as excellent targets for regenerative therapies^43–45^. In this regard, MGCs cultured under appropriate circumstances *in vitro*^46,47^ can, when transplanted *in vivo*^46,47^, display an improved ability to migrate towards retinal injured sites. In this sense, our results show that the migratory capacity of MIO-M1 cells induced by α_2_M* depends on the intracellular distribution and traffic of LRP1 to the PM, which may have special clinical connotations in the treatment of proliferative retinopathies.

## Methods

### Reagents

Alexa Fluor-488 and Alexa Fluor-594 NHS ester amine-reactive dyes, Lipofectamine 2000 and RNAiMax, EZ-Link Sulfo-NHS-SS-Biotin, Streptavidin agarose beads, Transferrin Alexa Fluor-594 conjugate, DQ-Red BSA, LysoTracker Red DND-99, and Nunc Lab-Tek II chamber slides were from Thermo Fisher Scientific, Buenos Aires, Argentina. Collagen type I, Recombinant human IGF-1, and wortmannin were purchased from Sigma-Aldrich (St. Louis, MO, USA). Mowiol 4–88 reagent was from Calbiochem (Merck KGaA, Darmstadt, Germany). Primary antibodies for immunofluorescence experiments; rabbit anti-LRP1 (ab92544), mouse anti-LRP1 (ab28320), rabbit anti-Rab4 (ab13252), rabbit anti-EEA1 (ab2900), and rabbit anti-Rab7 (ab137029) were purchased from Abcam (Cambridge, MA). Primary antibodies for immunoblotting; rabbit anti-Akt (9272) was from Cell Signaling Technology (Beverly, MA), rabbit anti-pAkt (07-789) was from Merck KGaA (Darmstadt, Germany), mouse anti-β-actin (A2228), rabbit anti-human α_2_M (HPA002265) and rabbit anti-hemagglutinin (HA) **(**SAB1306169) were from Sigma-Aldrich (St. Louis, MO). Secondary antibodies used were species-specific conjugated with Alexa Fluor-488 or Alexa Fluor-594 (Thermo Fisher Scientific) for immunofluorescence (diluted 1/800) or IRDye 800CW or IRDye 680LT (LI-COR, Lincoln, NE) for immunoblotting (diluted 1/10000). Predesigned human siRNA for Rab10 was purchased from Sigma-Aldrich (Sigma # SASI _Hs02_000348924) 21-mer (5′-GCAAAUGGCUUAGAAACAU[dT][dT]-3′). Silencer negative control #2 siRNA (cat. no. 4390847) was from Thermo Fisher Scientific. The GFP-Rab7 construct was kindly provided by Dr. Maria Isabel Colombo and the HA-GFP-mLRP4 construct was generated as previously described^31^. The α_2_M was purified from human plasma following a procedure previously reported^48^ and α_2_M* was generated by incubating α_2_M with 200 mM methylamine–HCl for 6 h at pH 8.2, as previously described^49^. The fluorescent α_2_M* conjugates were generated through controlled labelling reactions using Alexa Fluor-488 and Alexa Fluor-594 NHS ester amine-reactive dyes and following manufacture’s recommendations. Briefly, 1 mg α_2_M* (2 mg/mL) was incubated with the aforementioned dyes at a 5:1 molar ratio to α_2_M during 2 h, at room temperature (RT) and protected from light. Unreacted fluorophore was quenched with 0.15 M glycine, and α_2_M* conjugates were dialyzed against PBS in the dark at 4 °C for 3 days. The concentration of each conjugate was calculated by spectrophotometry and the degree of labelling was estimated using the mathematical formula provided by the manufacturer.

### Cell culture

The spontaneously immortalized human Müller cell line (MIO-M1) was kindly provided by Dr. G. Astrid Limb (University College London, Institute of Ophthalmology and Moorfields Eye Hospital, London, UK). This cell line has the same phenotypic and functional characteristics than a primary culture of Müller glial cells and expresses the same antigenic markers as well as has the same electrophysiological response to glutamate^50^. In this sense, the results obtained in MIO-M1 cells were first obtained in primary cultures of Müller cells freshly isolated from human retinas. Cells were cultured in DMEM-high glucose (4.5 mg/ml) with 2 mM L-glutamine (GlutaMAX; Gibco^®^, Invitrogen) and supplemented with 110 mg/ml sodium pyruvate, 10% (v/v) fetal calf serum (FCS) and 100 U/ml penicillin/streptomycin (Invitrogen) at 37 °C with 5% CO_2_.

For stimulations with α_2_M*, MIO-M1 cells were serum starved for 30 min in DMEM- high glucose and incubated at different time points with 60 nM α_2_M*. Cells were then washed three times on ice with either cold PBS supplemented with 1 mM Na3VO4 or cold PBS supplemented with calcium and magnesium, depending on whether they were processed for immunoblotting or immunofluorescence, respectively. For cell migration assays, MIO-M1 cells were stimulated with 60 nM α_2_M* or 10 nM IGF-1 for 12 h.

To silence Rab10 expression, cognate siRNA was delivered using Lipofectamine RNAiMax. Briefly, cells were transfected with either 5 pmol/well siRNA (for 96 wells plate) or 60 nM siRNA (for 6 wells plate) against scramble sequence or Rab10 for 6 h in Opti-MEM 1 × (Gibco®, Thermo Fischer Scientific) supplemented with 10% FCS. Cells were used within the next 24–48 h.

Transient transfections of GFP-Rab7 and HA-GFP-mLRP4 constructs were performed using Lipofectamine 2000 following manufacture’s recommendations. Cells were used 24–48 h after transfection.

### Immunoblotting

MIO-M1 cells were cultured in 6-well plates at 37 °C and after incubations with vehicle or α_2_M*, cell protein extracts were prepared using RIPA lysis buffer (50 mM Tris-HCl pH 8.0, 150 mM NaCl, 1% Tritón X-100, 0.5% Sodium deoxycholate, 0.1% SDS, 1 mM PMSF, 10 mM Sodium ortho-vanadate and protease inhibitor cocktails (Sigma-Aldrich)). Twenty micrograms of total protein were diluted in sample buffer 5X with DTT (dithiothreitol), boiled for 5 min, and separated by SDS-PAGE. Afterward, proteins were electrotransferred to a nitrocellulose membrane (GE Healthcare Life Science, Amsterdam), blocked with 5% nonfat dry milk in a Tris-HCl saline buffer containing 0.01% Tween 20 (TBS-T) for 60 min at RT, and incubated overnight with primary antibodies at 4 °C. Finally, the membranes were incubated with fluorescent secondary antibodies for 45 min at RT and developed using an *Odyssey* CLx near-infrared fluorescence imaging system (LI-COR Biosciences, Lincoln, NE). Results were acquired and quantified using the Image Studio 4.0 software (LI-COR). Representative images were processed using Adobe Photoshop CS4 (Adobe Inc.). Briefly, selected regions were cropped from full western blot images (shown in Supp. Fig. S3) and color images were converted to grayscale. Contrast or brightness adjustments were not performed.

### Biotinylation of PM proteins

To determine the level of LRP1 at the PM, MIO-M1 cells cultured in 6 wells plates were incubate for 2 h on ice with 0.1 mg/mL EZ-Link Sulfo-NHS-SS-Biotin solution with gentle rocking, followed by five washes and 45-min incubation on ice with 0.1 M glycine in PBS. Afterward, biotinylated cells were washed three times with cold PBS and lysed as previously described. Twenty micrograms of protein lysates were used as input, and 200 µg were incubated overnight with 65 µl of 50% slurry strepavidin-agarose beads for 2 h at RT. The centrifuged and pelleted beads were washed three times with 1% Triton-X100 in PBS. Finally, the biotinylated-PM proteins were eluted by adding sample buffer 1X with 1 M DTT, boiled for 5 min, and further analyzed by SDS-PAGE and immunoblotting.

### Cellular ELISA for detection of PM antigens

MIO-M1 cells, wild type or transiently transfected with HA-GFP-mLRP4 were cultured in 96-well plates as previously described. After incubations with vehicle or α_2_M* for different time points, the cells were washed with cold PBS, fixed with 4% (v/v) paraformaldehyde (PFA), quenched with 0.1 mM glycine, and blocked with 5% (v/v) horse serum for 30 min on ice. Then, cells were incubated with rabbit anti-LRP1 antibody (1/1000) for 1 h on ice, followed by three washes of 5 min each with ice-cold PBS and an incubation with goat anti-rabbit IgG IRDye® 800CW (LI-COR) secondary antibody (1/10,000) for 1 h on ice. The resulting fluorescence was measured using the *Odyssey* CLx near-infrared fluorescence imaging system. Quantifications were performed by densitometry using Image Studio Software. When necessary, cells were preincubated with 40 μM wortmannin for 30 min.

### Immunofluorescence and confocal microscopy

MIO-M1 cells on glass coverslips were treated with vehicle or α_2_M* as described above. After stimulation, cells were washed with PBS, fixed with 4% PFA in cytoskeleton stabilization buffer (10 mM PIPES pH 6.8, 100 mM KCl, 300 mM sucrose, 2 mM EGTA, and 2 mM MgCl2), and quenched with 50 mM NH_4_Cl. As needed, fixed cells were permeabilized for 30 min with 0.5% (v/v) saponin, blocked with 2% BSA, and incubated with the respective primary antibody (diluted from 1/100 to 1/250) for 1 h at 37 °C. All cells were subsequently washed with PBS and incubated with secondary antibodies for 45 min at 37 °C. Finally, coverslips were mounted using Mowiol 4–88. Slides were allowed to air-dry overnight and stored in the dark at −20 °C until examination. Images were acquired using an Olympus FluoView FV1000 or Olympus FluoView FV300 confocal laser scanning microscopes (Olympus, New York, NY) both controlled by FV10-ASW Viewer 3.1 software. The sampling density was defined applying the Nyquist-Shannon sampling theorem (https://svi.nl/NyquistCalculator) and after acquisition the images were processed for colocalization analysis using ImageJ software (National Institutes of Health, Bethesda, MD).

### Pulse-chase assays

Pulse-chase experiments (letting the ligand bind in the cold, then allowing it to internalize at 37 °C over time), were performed in MIO-M1 cells seeded on glass coverslips. Briefly, cells were serum-starved for 30 min and then incubated (pulse) on ice with pre-chilled solutions of 60 nM fluorescent α_2_M* conjugates (α_2_M*-AF-488 or α_2_M*-AF-594) in combination or not with 5 µg/mL Tf-AF-594 for 20 min. Afterward, cells were washed three times with cold PBS, rewarmed, and incubated (chase) in serum free medium at 37 °C for the indicated time points. Depending on the assays, after the chase the cells were washed either with cold PBS or cold 0.1 M glycine-PBS, pH 2.5 (acid wash solution) for 5 min. Finally, the cells were imaged or processed for immunofluorescence as previously described.

### Fluorescent recovery after photobleaching (FRAP)

MIO-M1 cells transiently transfected with mLRP4-GFP-HA were seeded on Nunc Lab-Tek II chamber slides. After 24 h, the cells were incubated with vehicle or α_2_M* as previously described. The FRAP experiments were performed in an Olympus FV300 confocal laser scanning microscope equipped with a 60 × PLAPON oil immersion/1.42 NA (Olympus, Japan) objective, a 488 nm Argon laser, and a temperature controller set at 37 °C. Photo-bleaching was performed using 3× optical zoom and 300 scans of a region of interest (ROI) of 40 × 70 pixels covering a specific peripheral region of the cell at 8 µs/pixel, 100% transmission of 488 nm laser line. Pre- and post-bleaching images (5 and 100 images, respectively) were acquired every 2.7 s with a 512 pixel × 512 pixel resolution using the same objective. FRAP experiments were performed on at least 10 cells per condition and repeated experiments at least twice. Individual FRAP measurement curves were averaged to get a single FRAP curve. Data analysis was performed as previously published by Zheng and collaborators^51^.

### Cell migration assays

Control (scramble siRNA) and Rab10-silenced MIO-M1 cells were seeded at 5 × 10^5^ cells/well on 6-well plates coated with collagen type I (10 μg/cm^2^) and cell migration was examined by a 2-dimensional wound-scratch assay. Briefly, after reaching 100% confluence, the cells were serum starved overnight and then, a straight lesion was created with a sterile 10-μl pipette tip in the center of the MIO-M1 cell monolayer. This technique produced a consistent wound devoid of cells of ~35 mm long x 400 μm wide. After washing three times with serum-free DMEM-high glucose without phenol red to remove cell debris, the cells were incubated in the same medium with vehicle, α_2_M* or IGF-1 for 12 h at 37 °C and 5% CO_2_. At selected times (0 and 12 h), 3 random images of the wound per condition were acquired using a charge-coupled device (CCD) camera (Nikon) on a bright-field microscope (Nikon TU-2000 inverted microscope; Nikon, Tokyo, Japan) equipped with a 10× objective (0.3 NA) and a temperature/gas controller. Cellular migration was quantified following a procedure previously described^52^. Each image defined an average area of the wound equivalent to 5 × 10^5^ ± 1 × 10^4^ μm^2^ recorded to t = 0 h. Cells invading this area were counted to t = 12 h, and the results were expressed as number of cells invading the wound.

### Statistical treatment of data

The quantification of the colocalization levels was performed using the JACoP plug-in from ImageJ^53^. At least 50 cells/condition were analyzed, and Manders’ coefficients were calculated^54^, averaged and statistically compared by Student’s t-test. P-values < 0.05 were considered significant. For immunoblotting and cellular ELISA assays, the data were expressed as mean ± SEM, and a one-way ANOVA and Student’s t-test were performed for statistical analysis using GraphPad Prism 5.0. P-values < 0.05 were considered significant.

## Supplementary information

Supplementary Figures 1, 2, and 3
