# Supplementary material for: Activated α2-Macroglobulin Regulates LRP1 Levels at the Plasma Membrane through the Activation of a Rab10-dependent Exocytic Pathway in Retinal Müller Glial Cells
Source: Sci Rep. 2019 Sep 13;9:13234. doi: 10.1038/s41598-019-49072-6 (PMC6744500; doi:10.1038/s41598-019-49072-6)

Supplementary Information

Activated α_2-Macroglobulin Regulates LRP1 Levels at the Plasma Membrane through the Activation of a Rab10-dependent Exocytic Pathway in Retinal Müller Glial Cells

Jaldín-Fincati Javier R, Actis Dato Virginia, Díaz Nicolás M, Sánchez María C, Barcelona Pablo F, and Chiabrando Gustavo A

Supplementary Figure 1. Fluorescent α_2M* conjugates move sequentially from EEA1-positive early endosomes to Rab7-positive late endosomes. Representative confocal micrographs (middle optical section planes) of MIO-M1 cells showing the intracellular distribution of a) α_2M*-AF-488 and EEA1-positive early endosomes; and b) α_2M*-AF-594 and Rab7-positive late endosomes, during pulse-chase experiments with chase time points at 0, 5, 10, 15, 30, and 60 min. Merge images show the colocalization of the fluorescent α_2M*conjugates with each specific intracellular marker (red or green as appropriate). The insets [1-12 in a) and 1-7 in b)] are 4x digital magnifications of the depicted regions in the cells (dashed-line-squares) in merge images. Three independent experiments were performed and at least 25 cells were analyzed per condition.

Supplementary Figure 2. α_2M* and transferrin show segregated internalization pathways in MIO-M1 cells. Representative confocal micrographs (middle optical section planes) of MIO-M1 cells showing the intracellular distribution of α_2M*-AF-488 and Tf-AF-594, during pulse-chase experiments with chase time points at 0, 5, 10, 15, 30, and 60 min. Merge images show the colocalization of both fluorescent proteins. The insets (1-11) are 4x digital magnifications of the depicted regions in the cells (dashed-line-squares) in merge images. The mask images depict colocalized pixels (white) in merge images and the mean of the Manders' coefficients with their respective standard deviations (MC±SD). Three independent experiments were performed and at least 50 cells were analyzed per condition.

Supplementary Figure 3. Uncropped western blots.

Supplementary Figure 1

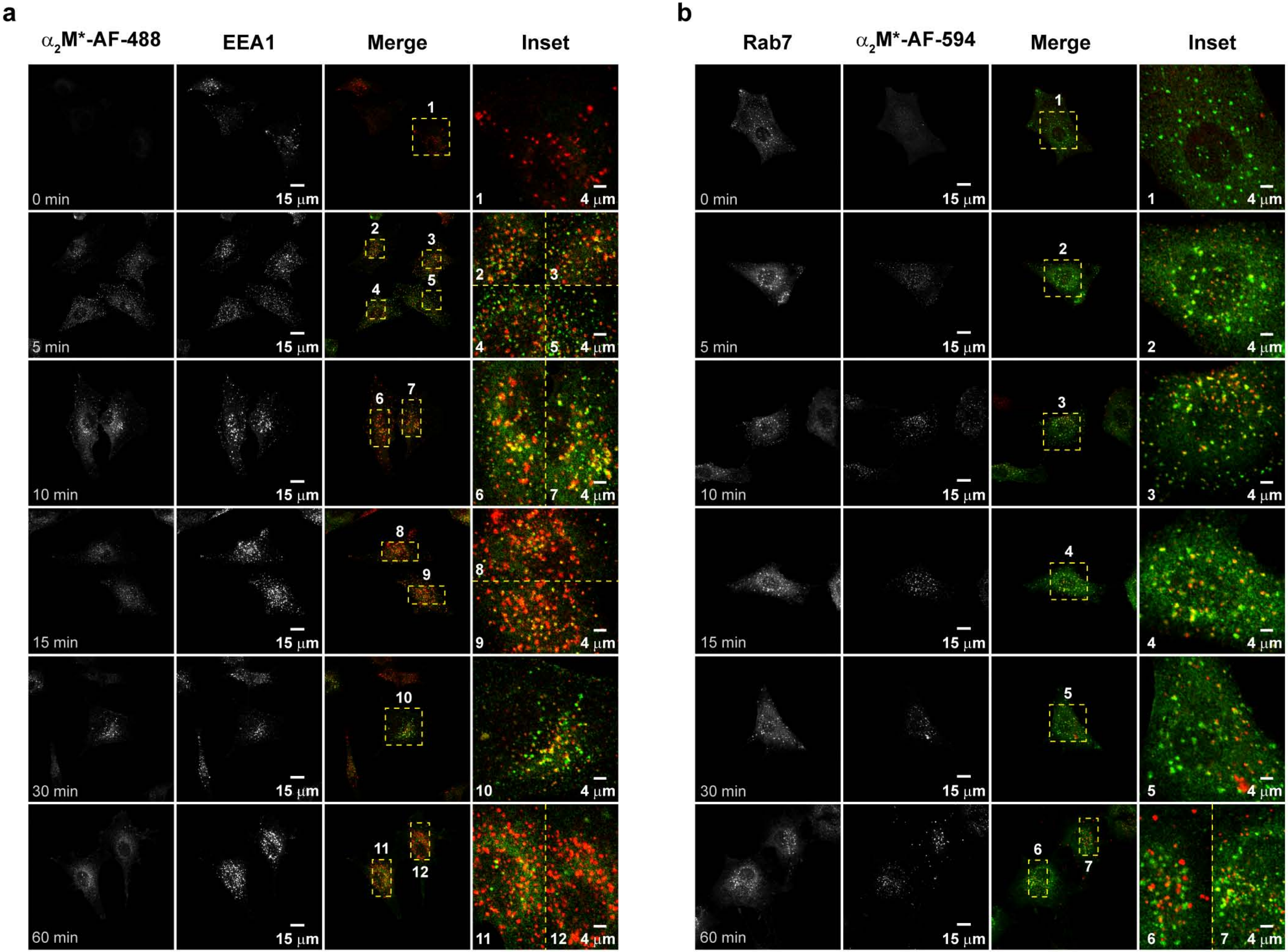

Supplementary Figure 2

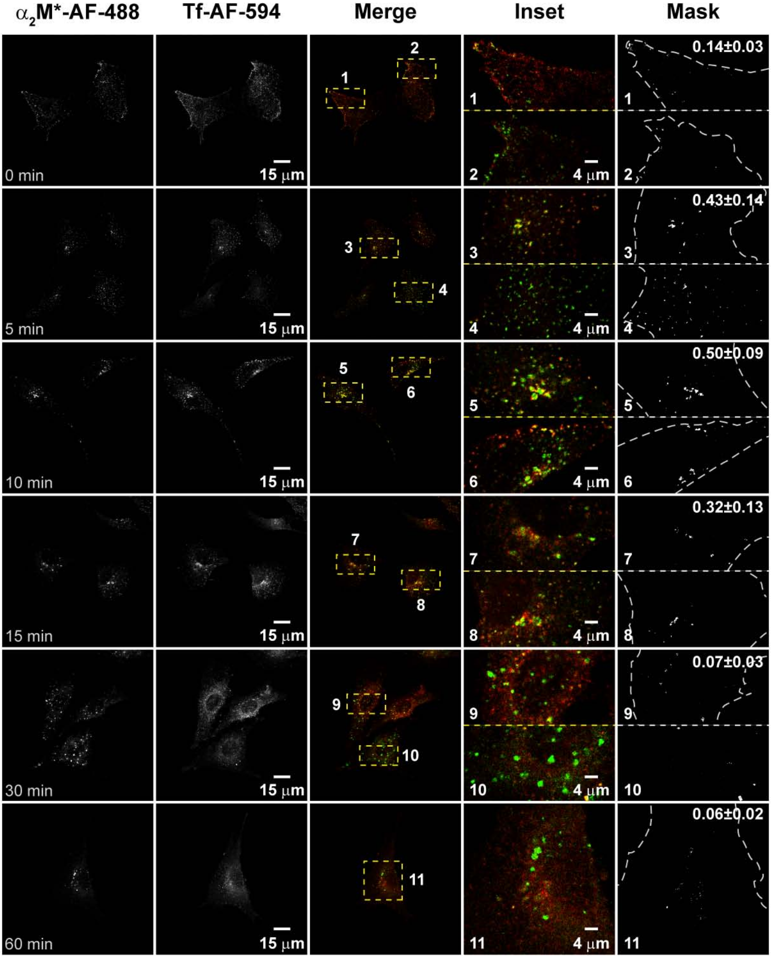

Supplementary Figure 3

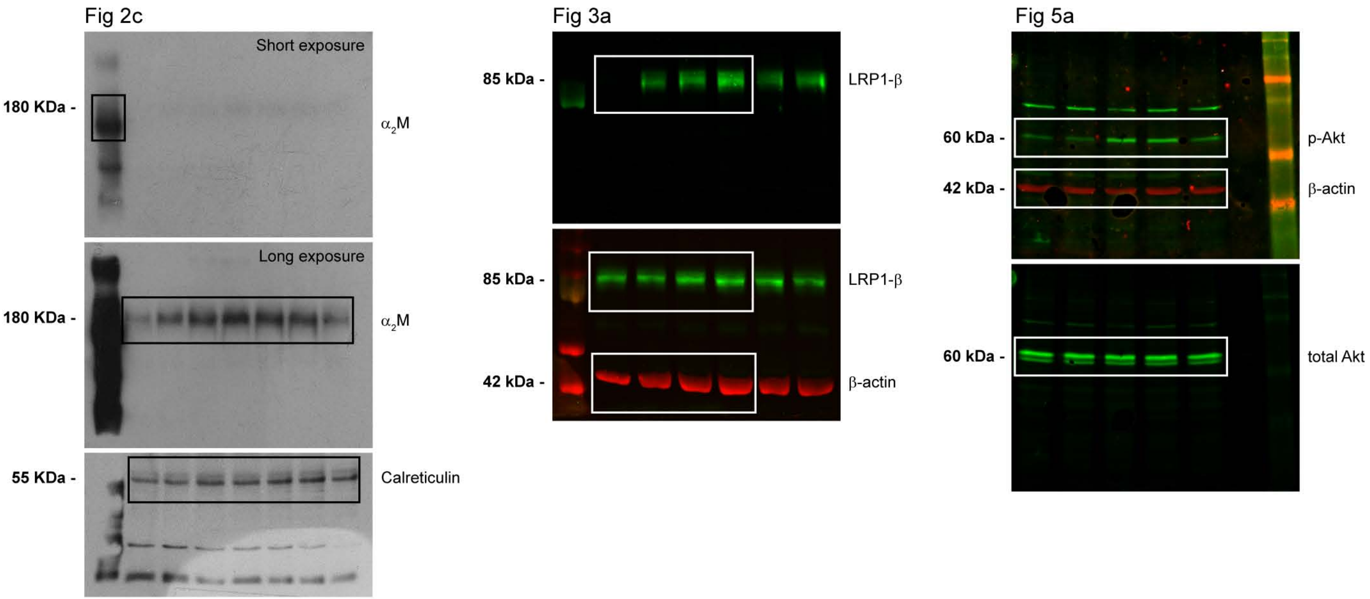

Supplement: Supplementary file 1 — Supplementary Figures 1, 2, and 3 [file 41598_2019_49072_MOESM1_ESM.pdf]
